# Central Slip Repair using Trans-articular K-wires: A Comparative Study.

**DOI:** 10.1016/j.jpra.2023.05.004

**Published:** 2023-06-02

**Authors:** S. Carr, PJ. O'Donoghue, A. Bowe, B. O'Ceallaigh, E. Siney, JL. Kelly

**Affiliations:** 1 Department Plastic & Reconstructive Surgery Galway University Hospital, GUH; 2 Department of Hand Therapy Galway University Hospital, GUH

**Keywords:** Central Slip Repair, Extensor injury, K-Wire, Repair

## Abstract

Central slip disruption may lead to PIP joint dysfunction causing significant morbidity. Existing evidence for any specific surgical management of these injuries is limited but does favor early mobilization of the PIP joint.

*Aim:* To assess the functional outcome in a cohort of patients undergoing central slip repair with internal K-wire proximal interphalangeal joint splinting and complete immobilization against those with external splinting only.

*Methods:* A single center retrospective analysis of all patients that underwent operative central slip repair in our institution over a 5-year period. Data were collected via the HIPE database and clinical notes. Data relating to demographics as well as range of motion, total active motion {(TAM) (TAM%)} score, and hand therapy rehabilitation type were analyzed.

*Results:* The study population was n = 44 patients. N = 33 patients were treated without a K-wire and n = 11 treated with a K-wire. There was a male predominance, 81.8% (n = 36). Mean age was 40.4 years. There was no significant difference in the mean TAM achieved at final measurement between the “no K-wire” and the “K-wire” treatment groups [no K-wire 202.1° (standard deviations (SD) 40.0) vs. K-wire 187.4° (SD 28.2), p = 0.208]. The “no K-wire group” achieved a mean TAM % of 78.0 (SD 11.4) and the “K-wire group” achieved a mean TAM % of 72.1 (SD 10.8); no statistically significant difference in mean scores was observed between groups.

*Conclusion*: Our study has shown comparable functional outcomes between those having complete joint immobilization with internal K-wire splinting and those that are externally splinted only following central slip repair.

## Introduction

Extensor tendon injuries are a commonly encountered hand injury, and functional outcomes can often be limiting.^1^^,^^2^ Functional limitation post-injury is often more severe when compared with flexor tendon injuries.^3^^,^^4^ Extensor tendon injuries are classified as per the zone of injury with zone III extensor injuries often involving the central slip (CS).^1^^,^^2^ The mechanism of injury can be from closed hyperflexion injuries resulting in avulsion of the CS or from open penetrating trauma to the digit where the tendon is cut. This can occur with or without concomitant bony injury of the phalanges. Closed injuries frequently occur in those participating in sporting activities.^5^ However, the incidence of CS injury, or zone III extensor tendon injury, is higher with open penetrating trauma compared with closed injuries.^2^

If left untreated, CS injuries can progress to a boutonniere deformity. As described by Massengill, the boutonniere deformity results from flexion at the proximal interphalangeal joint (PIPJ) and extension at the distal interphalangeal joint (DIPJ), caused by volar subluxation of the lateral bands when the CS is disrupted.^6^ Boutonniere deformity can result in significant morbidity for patients due to the impact on hand function.

The management of a CS injury is dependent on the nature of the injury. Closed injuries are often managed conservatively with extension splinting to immobilize the PIPJ.^1^^,^^7^ Open injuries require surgical management. Surgical techniques described involve direct repair of CS, use of bone anchors, tendon grafts, and bone tunneled suturing of the CS, among other techniques.^2^^,^^8^^,^^9^ Post-operative external splinting is indicated to immobilize the PIPJ once surgical repair of CS is performed.^7^ The concept of “internal splinting” was first described by Pratt, where a longitudinal K-wire was placed along the length of the finger to immobilize the digit following an extensor tendon repair.^10^ This technique has been refined to the use of shorter K-wires traversing only one joint to immobilize it following repair. This technique, which involves immobilizing the PIPJ in extension allowing the CS to heal prior to active mobilization and rehab, is favored by some surgeons. Contrary to this, some surgeons prefer to avoid trans-articular K-wires due to concerns regarding stiffness of the joint.

There is a paucity of literature comparing the functional outcomes for various surgical and non-surgical management techniques of CS injuries.^2^ Specifically, there is no consensus agreement on the use of trans-articular K-wire fixation of the PIPJ following CS repair or reconstruction. To our knowledge, there are no studies to date comparing functional outcomes following internal splinting with K-wire fixation of the PIPJ following CS repair or reconstruction and external thermoplastic splint following CS repair.

## Aim and Objectives

The aim of this retrospective cohort study was to compare the functional outcomes between patients who underwent CS repair or reconstruction with K-wire fixation of the PIPJ and patients who underwent CS repair or reconstruction with external splinting. Primary outcomes were to compare active finger range of motion measured at final hand therapy appointment pre-discharge, total active motion (TAM), and TAM%. Secondary outcomes were to compare the incidence of boutonniere deformity, extent of the extensor lag at the PIPJ, and day of discharge from hand therapy post-surgery.

## Methods

The study is a single-center retrospective cohort study based on a prospectively maintained database of all patients that underwent operative CS repair in our institution over a 5-year period between 2017 and 2021. An electronic case report form was designed to retrospectively collect as well as prospectively gather and maintain information on patient demographics, operative details, range of motion (ROM) measurements, TAM score, and post-operative complications. This database was maintained by the authors. Demographic details were recorded from a centralized hospital database and clinical notes. Operative details were recorded from the operative note; ROM measurements and TAM score were measured by hand therapists. The TAM score refers to the sum of the active MCP, PIP, and DIP arc of motion in degrees of an individual digit. The calculation of TAM was done using the Strickland-Glogovac formula.^12^ This calculation can then be compared with the TAM of the contralateral hand as a percentage (TAM%) or the norm of 260° and using the Strickland and Glogovac system of classification whereby TAM > 150° or 85%–100% of the contralateral finger or the norm of 260˚ is classified as excellent, good is > 70%–84% of the norm, fair > 50%–69% of the norm, poor < 50%.^12^ In this study, it is reported as a percentage of the norm of 260°. TAM is a validated measure and has been used previously in studies examining functional outcomes following CS injuries.^13^, ^14^, ^15^, ^16^

### Study population

Patients eligible for inclusion in the study were all patients with an acute acquired CS injury, open or closed, requiring surgical intervention that presented within 4 weeks of their initial injury. Patients excluded were those with existing boutonniere deformities, delayed presentation more than 4 weeks from initial injury (n = 8), multi-digit injuries (n = 1), those who underwent conservative management only (n = 6), and patients lost to follow up who did not have any functional outcome measurements recorded (n = 12). Forty-four patients met the inclusion criteria.

**Figure 1:**
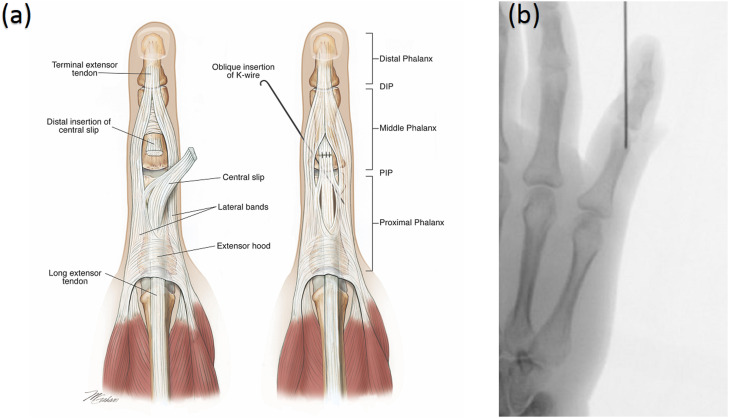
**(a)** Illustration of central slip repair and K-wire placement. **(b)** Plain radiograph of oblique trans-articular K-wire placement.

The cohort consisted of patients who underwent repair or reconstruction of the CS with or without internal splinting using a K-wire. For those who underwent internal splinting with K-wire fixation, a 1.1 mm K-wire was inserted at the time of repair traversing the PIP joint (Figure 1). The K-wire was left in situ for 4 weeks and was removed using a sterile technique as a local anesthetic procedure in a day surgery theater. All CS repairs and concomitant injured structures were repaired using a standard operative repair technique. The operative technique included a standard modified Kessler repair with some variability observed regarding the type and size of suture (4.0 or 3.0, PDS vs. Maxon) with four (n = 4) cases requiring the use of a Mitek bone anchor using the technique previously described in the literature by the lead author.^11^

Patients were followed up in the outpatient department at one week post operatively for wound review and application of a thermoplastic splint. Patients wore the splint continuously until 6 weeks post-surgery. K-wires were removed 4 weeks post-surgery. A graded flexion program and a splint weaning program were commenced at 6 weeks guided by hand therapy. Outcome measures were recorded at the final hand therapy appointment. Active ROM was measured in degrees with a finger goniometer using a standardized protocol. The same departmental protocol was used throughout when measuring the ROM to ensure intra-rater and inter-rater reliability. The same make and model of goniometer was used, and the positioning and verbal instruction were consistent for all patients.^16^^,^^17^ Once splint weaning was commenced, rehabilitation consisted of a gradual ROM and return to function and strength.

### Statistical analysis

Statistical analysis was performed using R statistical software package. Categorical data were described using counts and percentages. Differences in proportions were analyzed using Fisher's exact test. Where the distribution approximated normal, data were described using means and standard deviations (SD). Means were compared between groups using Welch's two-sample t-test. Where the distribution was not normal, data were described using medians and interquartile range (IQR), and groups were compared using the Wilcoxon rank sum test.

## Results

### Characteristics of the study population

The characteristics of the study population are described in Table 1. After applying the inclusion and exclusion criteria, the study population consisted of n = 44 participants, of whom 81.8% (n = 36/44) were male. The mean age was 40.4 years (SD 19.3). The index finger was the most frequently injured digit accounting for 34.1% (n = 15/44) of injuries, and the dominant hand was involved in 50.0% of cases. Laceration was the most prevalent mechanism of injury, and most injuries (88.6%) were open.

**Table 1:** Characteristics of the Study Population.

	Overall n = 44	No K-wire n = 33	K-wire n = 11	p-value
**Gender**				
Male	36 (81.8)	27 (81.8)	9 (81.8)	
Female	8 (18.2)	6 (18.2)	2 (18.2)	1.000*
**Age – n, mean (SD)**		40.9 (18.5)	39.1 (22.5)	0.815^
**Dominant hand involved**	22 (50.0)	15 (45.5)	7 (63.6)	0.488*
**Digit involved**				
Index	15 (34.1)	11 (33.3)	4 (36.4)	
Middle	11 (25.0)	10 (30.3)	1 (9.1)	
Ring	6 (13.6)	4 (12.1)	2 (18.2)	
Little	12 (27.3)	8 (24.2)	4 (36.4)	0.594*
**Mechanism**				
Crush	8 (18.2)	6 (18.2)	2 (18.2)	
Hyperflexion	4 (9.1)	3 (9.1)	1 (9.1)	
Laceration	32 (72.7)	24 (72.7)	8 (72.7)	1.000*
**Open injury**	39 (88.6)	30 (90.9)	9 (81.8)	0.586*
**Bone injury**	11 (25.0)	6 (18.2)	5 (45.5)	0.108*

⁎ Fisher's exact test

^ Welch's two-sample *t*-test

Of the 44 participants included in the study, n = 33 underwent CS repair or reconstruction with external splinting and are referred to as the “no K-wire group,” and n = 11 underwent CS repair or reconstruction with internal splinting using a K-wire and are referred to as the “K-wire group.” There were no statistically significant differences observed between the treatment groups with regard to gender, age, involvement of the dominant hand, digit involved, or mechanism of injury.

### Surgical repair and splinting regime

The type of surgical repair and splint used are described in Table 2. The majority of patients, 88.6% (n = 39/44), underwent repair or reconstruction of the CS alone, with n = 4 also having lateral band repair and n = 1 having repair of multiple structures. The most frequently used splint was a PIPJ circumferential splint. There were no statistically significant differences observed between the K-wire treatment groups regarding the type of surgical procedure or the splinting regime used.

**Table 2:** Surgical repair and splinting characteristics.

	Overall N = 44	No K-wire n = 33	K-wire n = 11	p-value
**Repair**				
Central slip (CS)	39 (88.6)	29 (87.9)	10 (90.9)	
CS + lateral band	4 (9.1)	3 (9.1)	1 (9.1)	
CS + multiple structures^	1 (2.3)	1 (3.0)	0 (0.0)	1.000
**Splint used**				
PIP DIP circumferential+	3 (6.8)	3 (9.1)	0 (0.0)	
PIP circumferential	39 (88.6)	28 (84.8)	11 (100.0)	
Hand-based extension	2 (4.5)	2 (6.1)	0 (0.0)	0.751

Fisher's exact test.

^ Multiple structures refer to neurovascular structures as well as soft tissue and tendon.

+ PIP, proximal interphalangeal; DIP, distal interphalangeal.

### Functional outcomes

The objectively measured ROM outcomes and TAM scores for each treatment group are described in Table 3. Both groups commenced discontinued splinting and commenced full mobilization at a mean time of 6 weeks. Final ROM measurements were taken at a median of 14.0 weeks for the no K-wire group and at median time of 17.0 weeks for the K-wire group, but this difference was not statistically significant.

**Table 3:** Comparison of functional outcomes between K-wire and no K-wire groups.

	No K-wire n = 33	K-wire n = 11	p-value
Week at full mobilization – n, mean (SD)	23, 6.4 (0.5)	7, 5.86 (0.7)	0.523*
Week of final measurement – n, median (IQR)	31, 14.0 (10.5)	11, 17.0 (14.0)	0.373^
TAM – n, mean (SD)	27, 202.1 (40.0)	9, 187.4 (28.2)	0.208*
TAM % – n, mean (SD)	27, 78.0 (11.4)	9, 72.1 (10.8)	0.186*
MCP extension – n, mean (SD)	22, 12.4 (5.4)	8, 13.4 (5.7)	0.673*
MCP flexion – n, mean (SD)	32, 80.6 (13.0)	11, 81.1 (8.1)	0.891*
PIP extension – n, mean (SD)	23, -11.6 (9.6)	7, -13.3 (11.5)	0.734*
PIP flexion – n, mean (SD)	32, 75.7 (24.5)	11, 63.9 (30.9)	0.269*
DIP extension – n, mean (SD)	33, 1.0 (10.1)	11, -2.5 (10.4)	0.348*
DIP flexion – n, mean (SD)	32, 40.5 (19.7)	11, 39.1 (17.2)	0.829*

⁎ Normal distribution. p-value calculated using Welch's two-sample t-test.

^ Non-normal distribution. p-value calculated using Wilcoxon's rank sum test.

There was no significant difference in the mean TAM achieved at final measurement between the “no K-wire” and the “K-wire” treatment groups (no K-wire 202.1° (SD 40.0) vs. K-wire 187.4° (SD 28.2), p=0.208). The “no K-wire group” achieved a mean TAM % of 78.0 (SD 11.4), and the “K-wire group” achieved a mean TAM % of 72.1 (SD 10.8), with no statistically significant difference in mean scores observed between the groups.

The incidence of complications is shown in Table 4. There were 3 complications recorded in the study population, n = 2 boutonniere deformities, both of which occurred in the “no K-wire” treatment group, and n = 1 non-union, which occurred in a patient who underwent K-wiring.

**Table 4:** Comparison of complications between K-wire and no K-wire treatment groups.

	Total N = 44	No K-wire n = 33	K-wire n = 11	p-value
Boutonniere deformity	2 (4.5)	2 (6.1%)	0 (0.0)	1.000*
Non-union	1 (2.3)	0 (0.0)	1 (9.1)	0.250*

⁎ p-value calculated using Fisher's exact test.

## Discussion

The primary objective of this retrospective cohort study was to compare mean TAM scores at final follow-up between patients who underwent CS repair/reconstruction with external splinting and patients who underwent CS repair/reconstruction with internal K-wire fixation. In this study, we found no statistically significant difference in mean TAM scores between those treated with and without K-wire fixation at a median follow-up time of 14.0 and 17.0 weeks, respectively.

A literature review by Geoghegan et al. has highlighted the paucity and quality of published data regarding the management of CS injuries, with only two prospective cohort studies being previously published and only nine studies in total being identified that fit the inclusion criteria for their literature review.^2^ This highlights the limit and amount of quality comparative data against which we can analyze our study. They have rated all papers they identified as being “fair to poor” quality also. The mean sample size of the papers they identified was n = 27 (SD 33 patients), and the demographic data from their papers referenced are comparable to the demographics of our cohort. Comparatively, our study has n = 44 patients, making this study quite large in the context of the current available literature. This literature review highlights the heterogeneity of data collected, post-operative management regimes, outcome measures used, follow-up and functional outcomes, and variety in follow-up time points. The current evidence base for management of CS injuries is limited, and the authors allude to the fact that much of the data regarding CS management are equivocal. Geoghegan et al. conclude that the majority of the literature would support early mobilization and not prolonged immobilization. Our results would counter this in that our data have shown that post-operative joint immobilization with a trans-articular K-wire for a 4-week post-operative period, with removal of the K-wire at week 4 and commencement of the hand therapy rehabilitation regime at no later than week 5, can achieve comparable functional outcomes with patients that are mobilized earlier. There was also a lower complication rate and progression to boutonniere deformity in our K-wire group.

The only study that is directly comparable to our study is a study by Evans.^17^ This comparative study looked at functional results following active post-operative management that consisted of mobilization versus immobilization in CS repair. Their study analyzed a cohort of 55 patients separated into two comparative groups. Group 1 (n = 33) patients that were immobilized in a splint for 3–6 weeks and Group 2 (n = 25) patients that were treated with a short arc of motion (SAM) initiated from days 2 to 11 post-operatively. Their outcomes that achieved most statistical significance between Group 1 and Group 2 were mean day of initiation of motion, day of discharge from therapy, TAM, and PIPJ extensor lag with the patients in the SAM group commencing therapy sooner, being discharged sooner, having a higher TAM score, and a lower degree of extension lag at the DIPJ compared with Group 1.^17^ Interestingly, they analyzed simple injuries separately, and, in this cohort, they found that Group 1 (immobilized) had an average TAM of 137° and a TAM% of 63% normal, with Group 2 (SAM) having an average TAM of 147° and a TAM% of 75% of normal. Comparatively, our TAM and TAM% scores were 202° and 78% (no K-wire) and 187° and 72% (K-wire), respectively, which using the classification applied by Evans et al. constitutes a “good” functional outcome, which is comparable to their results.^17^ We acknowledge that there is comparative heterogeneity between the measurement of outcomes and data between our study and those of Evans et al., which makes it difficult to draw any definitive conclusions here.

We have only identified one other paper that has analyzed the use of trans-articular K-wires in the surgical management of CS repairs in the literature. Mehdi et al.^8^ have described a technique of Mitek™ repair of CS injuries with the use of trans-articular K-wires for joint immobilization with a single trans-articular K-wire and splinting for 2 weeks followed by gradual active and passive mobilization exercises. They achieved good to excellent outcomes in eight patients with a ROM outcome (%) ranging from 100% to 50% in this cohort. Again, these data are comparatively different from the data collected in this study, but they show that the use of trans-articular K-wires for joint immobilization over a short post-operative period can lead to good functional outcomes.

Analysis of the reported functional outcomes within the literature using mean TAM as an outcome measure has shown some variability. Looking at specific studies regarding CS repair and rehabilitation, Feuvrier et al. showed a mean TAM of 160°, Maddy et al. a mean TAM of 175°, Pratt et al. a mean TAM of 237°, and Saldana et al. a mean TAM of 169°, respectively.^13^, ^14^, ^15^, ^16^ Comparing these TAM scores with our cohort of patients with TAM measurements of 202.1° for our no K-wire group and 187.4° for our K-wire group, although there is heterogeneity within these groups regarding intervention and postoperative management rehabilitation, both our K-wire and no K-wire groups have had good to excellent functional outcome TAM scores when compared with the published literature. This also highlights that the use of internal splinting using trans-articular K-wires achieves a comparatively good functional outcome. Another outcome measure assessed in our study showing comparable outcomes between our no K-wire and K-wire group was PIPJ extension lag at the last follow-up. This was comparable for both groups at 11.6˚ for the no K-wire group and 13.3˚ (SD 9.6) for the K-wire group (SD 11.5).

The limitations of the study include the small sample size with 33 participants in the “no K-wire” group and 11 participants in the “K-wire group.” Statistical tests may be compromised by the small numbers analyzed. This is a limitation seen in many of the studies in this area,^2^ and further research should focus on building large cohorts through either specialist centers or through multicenter studies.

## Conclusions

Our results add to the literature on this topic where a paucity of data exists to date. Statistically significant data from a larger prospective study would be required to advocate for a definitive change in practice. The results of this study we believe are of clinical use in that we have shown that there is utility in terms of functional outcome in using trans-articular internal splinting of the PIPJ following a CS repair.
